# Estimating the economic cost of carbapenem resistant Enterobacterales healthcare associated infections in Singapore acute-care hospitals

**DOI:** 10.1371/journal.pgph.0001311

**Published:** 2022-12-07

**Authors:** Yiying Cai, Grace S. R. Hoo, Winnie Lee, Ban Hock Tan, Joanne Yoong, Yik-Ying Teo, Nicholas Graves, David Lye, Andrea L. Kwa

**Affiliations:** 1 Programme in Health Services & Systems Research, Duke-NUS Medical School, Singapore, Singapore; 2 Department of Pharmacy, Tan Tock Seng Hospital, Singapore, Singapore; 3 Department of Pharmacy, Singapore General Hospital, Singapore, Singapore; 4 Department of Infectious Diseases, Singapore General Hospital, Singapore, Singapore; 5 Yong Loo Lin School of Medicine, National University of Singapore, Singapore, Singapore; 6 Research for Impact, Singapore, Singapore; 7 Saw Swee Hock School of Public Health, National University of Singapore, Singapore, Singapore; 8 Department of Infectious Diseases, National Centre for Infectious Diseases, Singapore, Singapore; 9 Department of Infectious Diseases, Tan Tock Seng Hospital, Singapore, Singapore; 10 Lee Kong Chian School of Medicine, Nanyang Technological University, Singapore, Singapore; 11 Singhealth Duke-NUS Medicine Academic Clinical Programme, Singapore, Singapore; 12 Emerging Infectious Diseases, Duke-National University of Singapore, Singapore, Singapore; Oxford University Clinical Research Unit Nepal, Patan Academy of Health Sciences, NEPAL

## Abstract

Quantifying the costs of hospital associated infections (HAIs) caused by carbapenem-resistant Enterobacterales (CRE) can aid hospital decision makers in infection prevention and control decisions. We estimate the costs of a CRE HAI by infection type and the annual costs of CRE HAIs to acute-care hospitals in Singapore. We used tree diagrams to estimate the costs (in Singapore dollar) of different CRE HAI types from the health service perspective and compared them to the costs of carbapenem-susceptible HAIs. We used two approaches to estimate costs–direct costs of consumables for infection prevention and treatment; and costs associated with lost bed days. Cost of a HAI were extrapolated to annual CRE HAI incidence in Singapore acute-care hospitals to estimate the annual cost to the hospitals. We found that the cost of a CRE HAI based on direct cost and lost bed days are SGD$9,913 (95% CI, SGD$9,431–10,395) and SGD$10,044 (95% CI, SGD$9,789–10,300) respectively. CRE HAIs are markedly higher than the carbapenem-susceptible HAIs for all infection types. In both approaches, CRE pneumonia was the costliest infection. Based on a CRE HAI incidence of 233 per 100,000 inpatient admissions, CRE HAIs costed SGD$12.16M (95% CI, SGD$11.84–12.48M) annually based on direct costs, and SGD$12.33M (95% CI, SGD$12.01–12.64M) annually based on lost bed days. In conclusion, we described the cost of CRE HAIs in Singapore hospitals and identified infections with the highest costs. The findings may be useful in informing future economic evaluations of competing CRE HAI prevention and treatment programmes.

## Introduction

Carbapenem resistant Enterobacterales (CRE) is a major global healthcare threat with substantial morbidity and mortality. Temkin et al. estimated that in 2014, carbapenem resistant *Escherichia coli* and *Klebsiella pneumoniae* caused 3.1 million serious infections worldwide [1]. In Singapore, the incidence of CRE clinical infections was estimated to range from 7.73 to 10.32 per 100,000 patient-days [2].

The rising incidence of CRE infections can place a financial burden on healthcare systems. Compared to the carbapenem-susceptible counterparts, CRE infections have higher healthcare costs. There is a need for contact precautions, length of stay will increase and there will be additional treatment with novel antibiotics and/or antibiotic combinations. Accurate cost estimates of infections provide information to influence policies on infection control and antimicrobial stewardship and provide incentive to improve diagnostic and treatment capabilities [3, 4].

Few studies have estimated the economic burden associated with CRE healthcare associated infections (HAIs) [4, 5]. Their findings may only be relevant to the specific country of study due to differences in healthcare systems and funding structure. In this study, we estimate the costs of a CRE HAI in Singapore to hospitals by infection type and compared it to a carbapenem-susceptible Enterobacterales (CSE) HAI. We also estimate the annual costs of CRE HAIs to acute care hospitals in Singapore.

## Methods

### Study setting and target population

In 2019 there were 10 public acute-care hospitals in Singapore comprising of general hospitals and specialty centres with acute-care inpatient facilities. These hospitals provide a variety of emergency and elective medical and surgical services, as well as specialty services including solid organ transplant, management of haematological and oncological malignancies, maternity and neonatal care services, and management of burns. A large proportion of the annual budget in Singapore public hospitals is provided by government block-based funding, which allocates a fixed amount for each average admission [6]. A smaller proportion of revenue arise from admitting private patients, which is capped [6]. All public hospitals have existing infection control practices in accordance to the national infection control guidelines and regulations, which includes active CRE surveillance and isolation of patients that are colonized or infected with CRE [7]. All public hospitals have established antimicrobial stewardship programmes [8]. The median bed occupancy rate in 2019 was 85.7% (bed occupancy rate range: 70.6%– 91.5%) [9].

The population modelled in this study is adult inpatients in Singapore public hospitals who acquired a HAI attributed to a microbiologically confirmed CRE. These patients were compared to adult inpatients in Singapore public hospitals with a HAI attributed to a microbiologically confirmed CSE (i.e., comparator group). HAIs were defined according to the European Centre for Disease Prevention and Control (ECDC) definition.

### Study perspective and design

**Fig 1:**
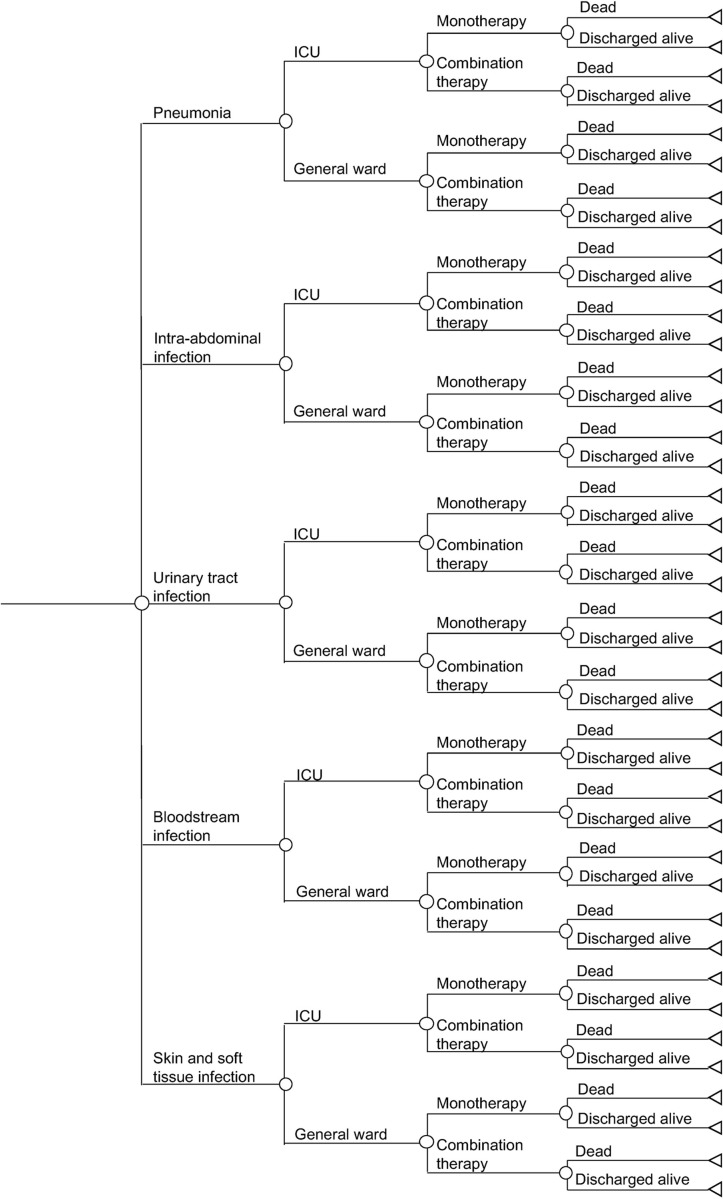
Tree diagram for carbapenem-resistant Enterobacterales infections.

**Fig 2:**
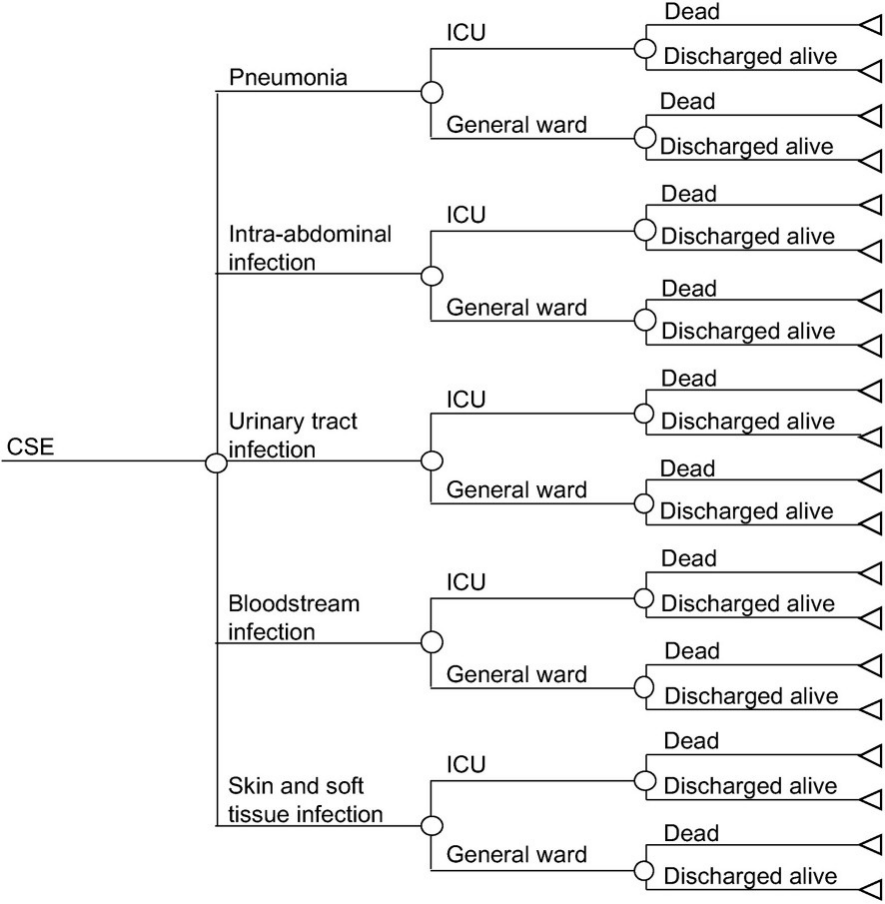
Tree diagram for carbapenem-susceptible Enterobacterales infections.

We conducted the cost analysis from health service perspective. We used a tree diagram to predict the costs of a CRE HAI for the duration of the infection within an inpatient admission (see Fig 1). Each patient enters the model with a probability of developing one of five HAI types: pneumonia; intra-abdominal infection; urinary tract infection; catheter-related bloodstream infection or other bloodstream infection of unknown source (collectively referred to as bloodstream infection); and skin and soft tissue infection, including superficial and deep tissue surgical site infections (collectively referred to as skin and soft tissue infection). Each HAI is associated with its own probability of intensive care unit (ICU) admission; patients with ICU admission were assumed to be admitted in the ICU for a range of 25%– 75% of the entire treatment duration. Each infection type, regardless of ICU or general ward, has its own probability of receiving monotherapy or combination antibiotic therapy. All patients were assumed to receive antibiotic treatment for the HAIs. Each patient then had a probability of in-hospital mortality, depending on the susceptibility profile, infection type and ward type. A similar tree diagram was used to model the costs of a CSE HAI, which served as a comparator, except patients with CSE HAIs only received monotherapy antibiotics (see Fig 2).

### Ethics

This study is waived from review by the SingHealth ethics review board as it employs only aggregate data derived from published studies.

### Data input

The input parameters for probabilities and outcomes were derived from a case-control study on CRE HAIs published by Hoo *et al*. (see Table 1) [10]. In the study, patients with CRE and CSE HAIs were indirectly matched for time at risk by matching to a common control group without infection. Estimates for annual CRE HAI incidence were based on studies published by Cai *et al*. [11, 12]. Drug doses followed international antibiotic guidelines [13]. For patients receiving antibiotic combination therapy, we assumed an equal probability of treatment for each of the various drugs to model the average across all the possible combinations. Meropenem and amikacin were selected as the carbapenem and aminoglycoside of choice respectively. Polymyxin B and colistin were respectively the intravenous and nebulized polymyxin of choice. All patients with CRE pneumonia were assumed to receive nebulized colistin as it is common practice to prescribe nebulized colistin to patients with pneumonia caused by carbapenem-resistant organisms in local acute-care hospitals [14, 15]. The frequency of routine tests and procedures were specific to the ward type; additional tests and procedures were infection specific (see Table 2). All assumptions in the model were applied after thorough review of local literature describing control and treatment of CRE infections. To validate the assumptions, two round-table meetings were held with local ID physicians, ID pharmacists and local epidemiologists.

**Table 1:** Model input for probabilities and outcomes [10].

Parameter	Estimate	Prior distribution (α,β)
CRE infections		
Probability of infection type		
Pneumonia	0.24	Beta (16.91, 54.30)
Intra-abdominal infection	0.29	Beta (20.69, 51.28)
Urinary tract infection	0.18	Beta (12.20, 57.53)
Bloodstream infection	0.11	Beta (7.56, 59.63)
Skin and soft tissue infection^*a*^	0.18	-
Probability of ICU admission		
Pneumonia	0.47	Beta (7.78, 8.78)
Intra-abdominal infection	0.17	Beta (3.05, 14.89)
Urinary tract infection	0.00	-
Bloodstream infection	0.33	Beta (2.53, 5.14)
Skin and soft tissue infection	0.53	Beta (6.93, 6.15)
Probability of combination antibiotic therapy		
Pneumonia	0.52	Beta (8.63, 7.97)
Intra-abdominal infection	0.35	Beta (6.97, 12.94)
Urinary tract infection	0.14	Beta (1.40, 8.59)
Bloodstream infection	0.45	Beta (3.59, 4.39)
Skin and soft tissue infection	0.40	Beta (5.19, 7.79)
Probability of mortality in ICU patients		
Pneumonia	0.78	Beta (5.51, 1.55)
Intra-abdominal infection	0.75	Beta (2.63, 0.88)
Bloodstream infection	0.33	Beta (1.06, 2.16)
Skin and soft tissue infection	0.62	Beta (4.38, 2.69)
Probability of mortality in general ward patients		
Pneumonia	0.40	Beta (3.49, 5.23)
Intra-abdominal infection	0.21	Beta (3.22, 12.10)
Urinary tract infection	0.00	-
Bloodstream infection	0.22	Beta (1.21, 4.29)
Skin and soft tissue infection	0.07	Beta (0.14, 1.88)
Time to death in days		
Pneumonia	12 (7.8)	Gamma (2.37, 5.07)
Intra-abdominal infection	9 (3.7)	Gamma (5.92, 1.52)
Bloodstream infection	8 (4.6)	Gamma (3.02, 2.65)
Skin and soft tissue infection	20 (11.0)	Gamma (3.31, 6.05)
Duration of treatment in days		
Pneumonia	39 (33.0)	Gamma (1.40, 27.92)
Intra-abdominal infection	27 (16.2)	Gamma (2.78, 9.72)
Urinary tract infection	15 (9.0)	Gamma (2.78, 5.40)
Bloodstream infection	18 (6.1)	Gamma (8.71, 2.07)
Skin and soft tissue infection	23 (11.2)	Gamma (4.22, 5.45)
CSE infections		
Probability of infection type		
Pneumonia	0.23	Beta (16.57, 55.49)
Intra-abdominal infection	0.18	Beta (12.84, 58.48)
Urinary tract infection	0.23	Beta (16.57, 55.49)
Bloodstream infection	0.16	Beta (10.97, 57.60)
Skin and soft tissue infection^*a*^	0.20	-
Probability of ICU admission		
Pneumonia	0.22	Beta (3.20, 11.33)
Intra-abdominal infection	0.08	Beta (0.72, 8.25)
Urinary tract infection	0.00	-
Bloodstream infection	0.15	Beta (1.40, 7.94)
Skin and soft tissue infection	0.06	Beta (0.58, 9.06)
Probability of mortality in ICU patients		
Pneumonia	0.21	Beta (2.94, 11.06)
Intra-abdominal infection	0.40	Beta (4.76, 7.14)
Bloodstream infection	0.27	Beta (3.17, 8.57)
Skin and soft tissue infection	0.25	Beta (3.63, 10.89)
Probability of mortality in general ward patients		
Pneumonia	0.25	Beta (3.99, 11.96)
Intra-abdominal infection	0.11	Beta (1.35, 10.91)
Urinary tract infection	0.00	-
Bloodstream infection	0.20	Beta (2.40, 9.58)
Skin and soft tissue infection	0.06	Beta (0.58, 9.06)
Time to death in days		
Pneumonia	7 (3.8)	Gamma (3.39, 2.06)
Intra-abdominal infection	17 (2.0)	Gamma (72.25, 0.24)
Bloodstream infection	9 (3.8)	Gamma (5.61, 1.60)
Skin and soft tissue infection	19 (8.7)	Gamma (4.77, 3.98)
Duration of treatment in days		
Pneumonia	11 (2.5)	Gamma (19.36, 0.57)
Intra-abdominal infection	24 (10.7)	Gamma (5.03, 4.77)
Urinary tract infection	13 (5.8)	Gamma (5.02, 2.59)
Bloodstream infection	18 (4.6)	Gamma (15.37, 1.18)
Skin and soft tissue infection	19 (8.7)	Gamma (4.77, 3.98)

^*a*^ The skin and soft tissue infections were not assigned a distribution in the probabilistic sensitivity analysis, but instead calculated using the formula: P_skin and soft tissue_ = 1- (P_pneumonia_ + P_intra-abdominal_ + P_urinary tract_ + P_bloodstream_), where P is the probability of each infection type in the probabilistic sensitivity analysis

Abbreviations used: CRE, carbapenem resistant Enterobacterales; CSE, carbapenem susceptible Enterobacterales; ICU, intensive care unit

**Table 2:** Model inputs and assumptions for costs.

Parameter	Estimate	Prior distribution (α,β)	Assumptions applied in model	Ref
Opportunity cost of a bed day				
ICU	902.06 (281.89)	Gamma (16.00, 56.38)	Patients with CRE in the general ward are admitted to single-bedded wards only as per local infection guidelines. Patients with carbapenem-susceptible Enterobacterales are distributed across the different bed types based on proportion provided by the Ministry of Health.	[12, 16]
Single-bedded	410.88 (128.40)	Gamma (10.24, 40.13)		
2-to-4-bedded	268.78 (83.90)	Gamma (10.26, 26.19)		
5-to-6-bedded	231.12 (84.00)	Gamma (7.57, 30.53)		
7-to-9-bedded	196.88 (61.53)	Gamma (10.24, 19.23)		
Direct costs–Contact precautions				
Contact precautions (per day)	56.79 (14.20)	Gamma (15.99, 3.55)	Incurred only by patients with CRE infections	Data from hospital database
Direct costs–Antibiotics (per day)				
Amikacin	29.80 (7.45)	Gamma (16.00, 1.86)	*Prescribed to CRE infections only as use of polymyxins and tigecycline are restricted to carbapenem-resistant infections. Levofloxacin was as the fluoroquinolone of choice for pneumonia; ciprofloxacin was the fluoroquinolone of choice for all other infection types. Tigecycline was only included as a treatment choice for monotherapy for intraabdominal infection and skin and soft tissue infections, but not for pneumonia, bloodstream infection and urinary tract infections. All patients with CRE pneumonia are prescribed nebulised colistin.	Data from hospital database
Amoxicillin-clavulanate	81.44 (20.36)	Gamma (16.00, 5.09)		
Cefepime	85.96 (21.49)	Gamma (16.00, 5.37)		
Ciprofloxacin	40.12 (10.03)	Gamma (16.00, 2.51)		
Colistin (nebulized)	100.85 (25.21)	Gamma (16.00, 6.30)		
Levofloxacin	147.80 (36.95)	Gamma (16.00, 9.24)		
Meropenem	122.15 (30.54)	Gamma (16.00, 7.63)		
Piperacillin-tazobactam	114.21 (23.37)	Gamma (16.00, 7.14)		
Polymyxin B*	93.50 (23.37)	Gamma (16.00, 5.84)		
Tigecycline*	93.50 (23.38)	Gamma (16.00, 13.80)		
Direct costs—Instrumentation and diagnostics (general)				
Biochemical panel (routine)	63.64 (15.91)	Gamma (16.00, 3.98)	All patients admitted to the ICU have daily biochemical and blood tests. Patients admitted in the general ward have biochemical and blood tests done once every three days. All patients admitted to the ICU are intubated and all intubated patients have daily blood gases and chest X-rays every two days. Patients with BSIs have three sets of blood cultures during treatment. All other patients have two sets of blood cultures. Patients with pneumonia have once weekly sputum cultures and chest X-rays (for non-ventilated patients). All UTI patients have one urinalysis and urine culture per week. All SSTI and intra-abdominal patients with one wound/tissue/fluid culture every seven days. All intra-abdominal infection patients have one CT scan per week. 50% of all SSTI patients with SSTIs require tissue debridement per week. 50% of all intraabdominal patients have one percutaneous drainage per week.	Data from hospital database
Blood panel (routine)	24.32 (6.08)	Gamma (16.00, 1.52)		
Blood gas	13.57 (3.39)	Gamma (16.00, 0.85)		
Blood culture (aerobic/anaerobic)	97.35 (24.34)	Gamma (16.00, 6.08)		
Chest x-ray	129.92 (32.48)	Gamma (16.00, 8.12)		
CT scan	1,014.38 (253.60)	Gamma (16.00, 63.40)		
Intubation (per day)	188.59 (47.15)	Gamma (16.00, 11.79)		
Percutaneous drainage of intra-abdominal collection	1,657.78 (414.45)	Gamma (16.00, 103.61)		
Sputum culture	48.41 (12.10)	Gamma (16.00, 3.03)		
Tissue/wound/fluid culture	48.41 (12.10)	Gamma (16.00, 3.03)		
Tissue debridement	29.18 (7.30)	Gamma (16.00, 1.82)		
Urinalysis	10.47 (2.62)	Gamma (16.00, 0.65)		
Urine culture	48441 (12.10)	Gamma (16.00, 3.03)		

Abbreviations used: CRE, carbapenem resistant Enterobacterales; CT, computerized tomography; ICU, intensive care unit; SSTI, skin and soft tissue infection; UTI, urinary tract infection

### Outcomes

We calculated HAI costs using two separate methods–direct costs; and opportunity costs associated with lost bed days. The direct costs tabulated costs that can be directly attributed to patient care including antibiotics, tests, and procedures, but excluded overhead and manpower costs (see Table 2) [17]. These direct costs represent costs that are potentially recoverable by the hospital for redirection to other purposes if the patient care service for the HAI was not rendered. Manpower costs were not included as such costs are committed and cannot be recovered in the short term even if HAI rates are reduced. As most of the cost data for drug treatment, tests and procedures were provided in terms of patient charges, we converted charges to costs using a ratio provided by the hospital. The opportunity cost of a lost bed day was estimated using the ‘cost accountant’ approach, whereby patient charges, prepared by the hospital accountants with the objective of cost recovery, were converted to costs using a cost-to-charge ratio [3]. All costs were adjusted to 2019 Singapore dollar (SGD) using a 5% inflation rate. Discounting was not applicable as costs were tabulated for the course of one hospital admission.

### Data analysis

All analyses were performed using TreeAge (Treeage Pro Healthcare 2021, Williamstown, MA, USA). Direct costs and opportunity costs associated with lost bed days for a single HAI (CRE or CSE) were reported for each infection type and overall. Data on a single CRE were combined with the estimated number of nosocomial CRE infections annually to provide estimates of the economic burden of CRE HAIs to Singapore hospitals. All costs were reported in Singapore dollars, as mean and 95% confidence interval (1 SGD = 0.75 USD). Uncertainties around the input variables were modelled using Monte Carlo simulations comprising of 1000 trials. Length of stay and costs were assigned the gamma distribution. Probabilities were assigned the beta distribution.

## Results

### Expected annual burden of CRE HAIs

It was previously estimated that the incidence of HAIs among patients admitted in public acute care hospitals in Singapore was 15,980 ± 840 patients per 100,000 inpatient admissions [12]. Based on the Singapore point prevalence survey on HAIs, 151 out of 727 HAIs (20.8%) were attributable to Enterobacterales; of these, 7.0% were non-susceptible to carbapenems (% of CRE HAIs out of all HAIs = 1.5%) [11]. Hence, we estimated that the incidence rate of CRE HAI is 233 ± 12 patients per 100,000 inpatient admissions, and a total of 1,224 ± 52 adult inpatients had CRE HAIs in Singapore public acute care hospitals in 2019 [16].

### Direct costs of a CRE HAI and CSE HAI

The direct costs of CRE HAIs are shown in Table 3. The mean direct cost of a CRE HAI and CSE HAI was SGD$9,913 (95% CI, SGD$9,431–10,395) and SGD$2,665 (95% CI, SGD$2,624–2,705) respectively (see Table 3). The cost of consumables varied depending on the HAI type. Across the different CRE HAI types, pneumonia and intra-abdominal infection were the costliest. Compared to a carbapenem-susceptible infection of the same type, pneumonia and skin and soft tissue infections were substantially costlier for CRE.

**Table 3:** Estimated cost of a single CRE-HAI and CSE-HAI based on direct costs and lost bed days.

	Mean cost per case based on direct costs (95% CI)		Mean cost per case based on lost bed days (95% CI)	
	CRE HAI	CSE HAI	CRE HAI	CSE HAI
All HAIs	9,913 (9,431–10,395)	2,665 (2,624–2,705)	10,044 (9,789–10,300)	4,680 (4,589–4,772)
Pneumonia	15,057 (14,324–15,789)	1,639 (1,617–1,660)	17,537 (16,612–18,461)	2,939 (2,885–2,992)
Intra-abdominal infection	10,110 (9,774–10,446)	8,676 (8,462–8,891)	9,359 (8,971–9,746)	6,789 (6,692–7,065)
Urinary tract infection	2,530 (2,441–2,619)	1,338 (1,305–1,371)	6,219 (5,960–6,478)	3,582 (3,474–3,690)
Bloodstream infection	4,267 (4,172–4,362)	1,898 (1,869–1,926)	7,669 (7,475–7,864)	4,697 (4,606–4,788)
Skin and soft tissue infection	6,936 (6,652–7,220)	2,127 (2,073–2,180)	10,832 (10,375–11,288)	5,407 (5,253–5,562)

Abbreviations used: CRE, carbapenem resistant Enterobacterales; HAI, healthcare associated infection

### Costs of a CRE HAI and CSE HAI based on lost bed days

The mean cost of a CRE HAI based on bed days lost was SGD$10,044 (95% CI, SGD$ 9,789–10,300) (see Table 3). Across the different CRE HAI types, pneumonia, skin and soft tissue infection and intra-abdominal infection were the costliest. In contrast, the cost of a single CSE HAI based on bed days lost was SGD$4,680 (95% CI, SGD$4,589–4,772). The cost of a CRE pneumonia was substantially higher when compared to the carbapenem-susceptible infection, where the cost of a CRE pneumonia was almost six times of a CSE pneumonia based on lost bed-days.

### Annual costs of CRE HAIs

Extrapolating the annual incidence of CRE HAIs to the infection costs, CRE HAIs costed SGD$12.16M (95% CI, SGD$11.84–12.48M) annually based on direct costs, and SGD$12.33M (95% CI, SGD$12.01–12.64M) annually based on lost bed days (Table 4). Intra-abdominal infection was the HAI type with the greatest total annual direct costs, with more than half of the direct costs attributable to instrumentation and procedure. Skin and soft tissue infections had the greatest total annual cost when estimated using bed days lost, followed by intra-abdominal infection and pneumonia.

**Table 4:** Burden and costs of CRE HAI in million (SGD) annually.

	Mean annual burden of CRE HAIs (95% CI)	Mean annual direct costs (95% CI)				Mean annual cost based on bed days (95% CI)
		All direct costs	Contact precaution	Antibiotics	Instrumentation and procedure	
All HAIs	1,227 (1,224–1,230)	12.16 (11.84–12.48)	1.77 (1.72–1.82)	5.52 (5.35–5.69)	4.87 (4.74–4.99)	12.33 (12.01–12.64)
Pneumonia	137 (134–140)	2.11 (1.99–2.24)	0.28 (0.27–0.30)	1.25 (1.18–1.33)	0.58 (0.54–0.62)	2.43 (2.28–2.57)
Intra-abdominal infection	357 (353–361)	3.61 (3.48–3.74)	0.45 (0.43–0.46)	0.89 (0.85–0.92)	2.28 (2.19–2.36)	3.32 (3.18–3.47)
Urinary tract infection	214 (211–218)	0.54 (0.52–0.56)	0.18 (0.17–0.19)	0.29 (0.28–0.30)	0.07 (0.07–0.07)	1.33 (1.27–1.39)
Bloodstream infection	136 (133–139)	0.58 (0.56–0.60)	0.13 (0.13–0.14)	0.27 (0.27–0.28)	0.18 (0.17–0.18)	1.06 (1.03–1.10)
Skin and soft tissue infection	382 (375–389)	2.64 (2.52–2.76)	0.48 (0.46–0.50)	1.20 (1.15–1.26)	0.96 (0.91–1.01)	4.17 (3.97–4.37)

Abbreviations used: CRE, carbapenem resistant Enterobacterales; HAI, healthcare associated infection

## Discussion

CRE HAIs are associated with considerable human and economic cost [18]. In this study, we estimated the cost of CRE HAIs to public hospitals in Singapore based on direct expenditure and lost bed days. We found that a CRE HAI more than costed three times of a CSE HAI based on direct costs; and two times based on lost bed days. Annually, CRE HAI costed Singapore acute care hospitals SGD$12 million based on both direct costs and lost bed days.

The main pillars for CRE HAI prevention include active surveillance, infection control and prevention, and antimicrobial stewardship [19]. A local study suggested that antimicrobial stewardship may be more important in prevention of mutations or genetic reassortment of CSE, while infection control measures (e.g., hand hygiene, environmental hygiene, and early isolation of CRE carriers) is crucial in preventing clonal bacterial spread or horizontal gene transfer [20]. The authors advised that efforts invested in antimicrobial stewardship versus infection prevention and control should be tailored to the local CRE epidemiology, taking into account the resistance mechanisms and modes of CRE transmission/acquisition [20]. In our study, we provided the cost of a CRE HAI as well as a CSE HAI from the health system perspective. The cost of a CRE HAI represented the amount incurred by the hospital if a CRE HAI had developed in an uninfected patient (e.g. *via* patient-to-patient or environment-to-patient transmission) [21]. In patients that developed a CRE HAI after a CSE HAI (e.g. *de novo* mutations or acquisition of plasmids harbouring carbapenemase genes, resulting in development of carbapenem resistance), the costs incurred by the hospital for the HAI episode would be the sum of the CRE HAI and CSE HAI. We believe that our cost data will be useful in informing future cost effectiveness analyses that consider the different modes of CRE HAI development/transmission.

We estimated the cost of CRE HAIs to Singapore hospitals using two approaches. The direct cost approach represented resources that may be given to other patients if CRE HAI rates were reduced [17]. To estimate the resources incurred, we applied assumptions for infection control and treatment similar to the methods used by Bartsch et al. [4]. We did not include manpower costs as such costs are not recoverable in the short term even if infection rates are reduced [22]. In our second approach, we used the cost of bed days used up to describe the costs of a CRE HAI. This approach implicitly assumed that each hospital is operating at close to full capacity. Hence, bed days released from the reduction of CRE HAI rates would be used for alternative health-producing activities that have a positive economic value, such as the monetary value that is derived from admitting patients who are currently on the waitlist for elective surgeries [23]. Our assumption is valid given that bed occupancy rates in Singapore public hospitals are often in excess of 85–90% [9]. We employed accounting costs as a surrogate measure of the economic opportunity cost of a bed day in Singapore. This is less than ideal as an economist seeks the value gained from the resources had they been used for an alternative purpose, but the hospital accountant is concerned with budgeting and cost recovery to ensure that the hospital stays financially viable in the future. Hence, costs derived from the cost-accountant method may not accurately represent the economic opportunity cost of a bed-day, which is often dependent on factors such as bed occupancy rates and waiting lists for elective procedures [3, 23, 24]. Costs derived from contingent valuation would have been more useful for decision-making but obtaining accurate estimates can be challenging [23].

To estimate the annual cost of infections, accurate measurements in the infection incidence and the additional length of stay is important [3, 24]. Our estimates for annual burden of CRE HAIs were based on a national point prevalence survey for HAIs [11, 12]. The study is the only nationwide HAI prevalence study in Singapore to date and employed rigorous methods for the survey conduct [11]. However, we acknowledge that infection control policy changes have changed since the COVID-19 pandemic. Newer surveys conducted after the onset of the pandemic may more accurately reflect the current burden of CRE HAIs in Singapore. Our estimates for excess length of stay were derived from a local study by Hoo et al, which addressed the time-varying nature of infections by matching for time to infection [10, 25]. However, it should be noted that while patients with CRE HAIs and CSE HAIs were indirectly matched for time at risk in the study, patients with CRE HAIs appeared to have higher prior ICU stay and antibiotic exposure. This may have confounded the excess mortality and length of stay findings reported in in the study [10]. We did not consider outcomes from other local studies as previous studies employed only time-fixed methods or did not clearly distinguish between community-onset and hospital CRE infections [2, 26–28].

The top two CRE HAIs with the greatest annual hospital costs in Singapore were intra-abdominal infections and skin and soft tissue infections. Targeting future investments in infection prevention against these infections may represent a good use of healthcare resources. Investing efforts in preventing CRE pneumonia will also be rational given the high excess cost per case compared to the CSE counterparts. Our cost estimates were conservative. We did not consider costs beyond the current hospitalization such as subsequent outpatient visits and outpatient antibiotic therapy. We also did not model the effects of further antibiotic resistance (e.g. polymyxin B resistance), which would have invariably resulted in greater costs to the hospitals.

This study has limitations. Firstly, our costs estimates for the direct costs were based on a cost-to-charge ratio, which may not accurately represent the true costs of the consumables due to variations in mark-ups across items [29]. Furthermore, our estimates for direct costs were based on assumptions, as opposed to a patient-level micro-costing approach. Data obtained from a patient-level costing approach would have more accurately represented the resources that were freed up from reduction of CRE HAI rates [24]. Next, our estimates only considered costs from the hospital’s perspective. The costs generated from our study will be informative for hospital decision makers and administrators for the development of hospital policies on CRE management [24]. However, such data may be less useful for national policy makers who more often require the societal costs of a CRE HAI for nation-wide policy making [24].

## Conclusions

Escalating healthcare costs are a major concern in health care delivery. Formal economic analyses must be conducted to provide objective evidence on cost-effectiveness of interventions. This study described the cost of CRE HAIs using a variety of measures and identified the CRE HAIs with the highest attributable costs and annual burden in Singapore. The findings will be useful in informing future economic evaluations of competing CRE prevention and treatment programmes in the hospitals.
